# HIF-1α as a Mediator of Insulin Resistance, T2DM, and Its Complications: Potential Links With Obstructive Sleep Apnea

**DOI:** 10.3389/fphys.2020.01035

**Published:** 2020-09-09

**Authors:** Agata Gabryelska, Filip Franciszek Karuga, Bartosz Szmyd, Piotr Białasiewicz

**Affiliations:** Department of Sleep Medicine and Metabolic Disorders, Medical University of Łódź, Łódź, Poland

**Keywords:** insulin resistance, T2DM2, OSA, hypoxia, HIF-1α

## Abstract

Obstructive sleep apnea syndrome (OSA) is described as an independent risk factor for the onset and progression of type 2 diabetes (T2DM), as well as for insulin resistance (IR). The mechanisms underlying these processes remain unclear. One of the proposed molecular mechanism is based on the oxygen-sensitive α-subunit of hypoxia-inducible factor 1 (HIF-1α)—a key regulator of oxygen metabolism. The concept that stabilization of HIF-1α may influence T2DM and IR is supported by cell and animal models. Cell culture studies revealed that both glucose uptake and glycolysis are regulated by HIF-1α. Furthermore, animal models indicated that increased fasting glucose may be caused by a single night with intermittent hypoxia. Moreover, in these models, hypoxia time was correlated with IR. Mice models revealed that inhibition of HIF-1α protein may downregulate fasting blood glucose and plasma insulin level. Administration of superoxide dismutase mimetic resulted in inhibition of HIF-1α protein, catecholamines, and chronic intermittent hypoxia-induced hypertension in a mice model. The hypothesis that hypoxia is an independent risk factor for IR is strengthened by experimentally confirmed improvement of insulin sensitivity among OSA patients treated with the continuous positive airway pressure. Furthermore, recent studies suggest that HIF-1α protein concentration is increased in individuals with OSA. In this literature review, we summarize the current knowledge about HIF-1α in OSA in relation to the possible pathways in which they contribute to metabolic disorders.

## Introduction

Obstructive sleep apnea (OSA) constitutes a rapidly growing health problem in the modern world (Gabryelska and Białasiewicz, 2020). Recent data suggest that the moderate and severe form of this disorder affect between 6 and 17% adults in the general population (Senaratna et al., 2017), while some research suggest that its prevalence is up to 23% among women and 49% among men (Heinzer et al., 2016). This trend may be an effect of increasing frequency of overweight/obesity, which is one of the strongest modifiable OSA risk factors. Interestingly, this dependency is bidirectional: BMI increment leads not only to higher OSA frequency but also to more severe course of this disease, whereas frequent sleep disruptions result in weight gain accompanied by poorer metabolic outcome (Farr and Mantzoros, 2017).

Numerous studies revealed connection between OSA and abnormal glucose metabolism [insulin resistance (IR), onset and progression of type 2 diabetes (T2DM)] (Morgenstern et al., 2014; Anothaisintawee et al., 2016). Additionally, Bulcun et al. (2012) observed that progression from snoring and/or mild OSA to severe OSA led to increased frequency of abnormal glucose metabolism. Thus, they suggested regular examination of possible glucose metabolism derangements among OSA patients, especially those with severe OSA (Bulcun et al., 2012). Moreover, they noted that subjective daytime sleepiness indices were independent risk factors of IR and T2DM (Bulcun et al., 2012). It is assumed that IR and T2DM in this group are related to recurrent tissue hypoxia (Drager et al., 2013). This hypothesis is supported by the observation that relatively mild, but intermittent desaturations were independent risk factors for metabolic dysfunction (Stamatakis et al., 2008; Drager et al., 2013). OSA is linked not only with IR/T2DM frequency but also with their severity, namely, OSA severity positively correlated with deterioration of T2DM outcomes (Farr and Mantzoros, 2017). Moreover, one of the most common OSA complications/comorbidities is a metabolic syndrome. Interestingly, a meta-analysis revealed that OSA predicted risk of metabolic syndrome, independently of obesity (Mesarwi et al., 2015; Qian et al., 2016); Coughlin et al. (2004) observed that metabolic syndrome was over nine times more likely to be present among OSA patients.

Although the relationship between OSA and metabolic disorders is intensively analyzed nowadays and OSA is described as an independent risk factor for onset and progression of T2DM and IR, the mechanisms underlying these processes remain not fully elucidated. Better understanding of this link may lead to a more efficient diagnostic process, as well as facilitate personalized treatment strategy (Mihaicuta et al., 2017; Carberry et al., 2018). Possibly underlying mechanisms include hypoxia, sleep fragmentation, inflammation, and oxidative stress, hormonal changes or increased sympathetic tone (Mesarwi et al., 2015; Farr and Mantzoros, 2017). In this minireview, we aim to analyze the link between metabolic complications of OSA and hypoxia in the context of hypoxia-inducible factors (HIFs). The importance of this choice is highlighted by publications supporting the central role of hypoxia in OSA-related comorbidities and possible severity biomarkers among OSA patients (Vavougios et al., 2014, 2016; Natsios et al., 2016).

## The Molecular Biology of Hypoxia-Inducible Factor

Hypoxia-inducible factor is a heterodimer composed of two units: α-subunit, which is oxygen-regulated, and constitutively expressed β-subunit (Semenza et al., 1997), belonging to helix-loop-helix Per/Arnt/Sim transcription factor family. To date, three analogs of HIF α-subunits are known (HIF-1α, HIF-2α—established regulatory factors; HIF-3α—uncertain role). The first one, HIF-1α, is the best-examined HIF α-subunit. Although its transcriptional level remains stable, HIF-1α protein is highly unstable under normoxia conditions (Wang et al., 1995), which entails the presence of oxygen-dependent degradation domain. Its low half-life time under normoxia condition, hydroxylation, and acetylation of oxygen-dependent degradation domains lead to its association with pVHL E3 ligase complex resulting in its degradation in the ubiquitin-proteasome pathway (Ke and Costa, 2006; Badawi and Shi, 2017). Upon post-translational stabilization under hypoxia conditions, active dimeric protein complex is transported to nucleus, wherein it binds hypoxia-response elements located in gene promoters, affecting expression of over 100 genes (Semenza, 2001; Masoud and Li, 2015; Wen et al., 2019; Gabryelska et al., 2020a). As hypoxia occurs in tissues with high proliferation rate (Czarnecka et al., 2019), HIF-1α is widely described in carcinogenesis pertaining to upregulation of genes involved in angiogenesis as well as proliferation.

On the other hand, under hypoxia, HIF is responsible for reprogramming of metabolic pathways (Rankin et al., 2009). HIF-1α is crucial for many physiological and pathological processes by controlling expression of genes involved in glucose metabolism, erythropoiesis/iron metabolism, vascular resistance, and circadian rhythm. The impact of HIF-1α on glucose metabolisms is described in relation to glucose uptake, glycolysis, as well as regulation of the tricarboxylic acid cycle (TAC). Genes mediating these processes, which are affected by HIF, were collected in Table 1. Considering effects of intermittent hypoxia present in OSA patients, one of the proposed molecular mechanisms of IR and T2DM is based on HIF-1 molecule.

**TABLE 1:** HIF-1 regulated genes involved in glucose metabolism.

Gene	Abbreviation	Source
Aldolase-A	*ALDA*	Semenza et al., 1996
Aldolase-C	*ALDC*	Semenza et al., 1996
Carbonic anhydrase-9	*–*	Wykoff et al., 2000
Eldolase-1	*ENO1*	Semenza et al., 1996
Glucose transporter 1	*GLUT1*	Chen et al., 2001
Glucose transporter 3	*GLUT3*	Chen et al., 2001
Glucose transporter 4	*GLUT4*	Sakagami et al., 2014.
Glyceraldehyde phosphate dehydrogenase	*GAPDH*	Graven et al., 1999
Hexokinase 1	*HK1*	Soni and Padwad, 2017
Hexokinase 2	*HK2*	Soni and Padwad, 2017
Lactate dehydrogenase A	*LDHA*	Semenza et al., 1996
Pyruvate dehydrogenase kinase 1	*PDK1*	Kim et al., 2006
Pyruvate kinase M	*PKM*	Semenza et al., 1994
Phosphofructokinase L	*PFKL*	Semenza et al., 1994
Phosphoglycerate kinase 1	*PGK1*	Semenza et al., 1994
6-phosphofructo-2-kinase/fructose-2,6- bisphosphate-3	*PFKFB3*	Minchenko et al., 2002
Ras-Related Protein Rab-20	*RAB20*	Hackenbeck et al., 2011
Thioredoxin interacting protein	*TXNIP*	Li et al., 2015

## Impact of HIF-1α on IR and T2DM in Cell Culture Models

Cell culture studies indicate that HIF-1α regulates both glucose uptake and glycolytic enzyme activity, significantly enhancing the process of glycolysis (Nagao et al., 2019). HIF-1α also plays a role in the downregulation of TAC (Kim et al., 2006). Overexpression of HIF-1α in cells under hypoxic conditions makes them secrete more lactate (Sato et al., 2014). In three cell types, HT1080 (fibrosarcoma), HepG2 (hepatoma), and HeLa (cervical carcinoma), the induction of glucose transporter 1 (GLUT-1) mRNA was measured after exposure to an atmosphere of 1% (hypoxia) and 21% (normoxia) oxygen for 16 h. Cells under hypoxia transcribed three- to fivefold more *GLUT-1* mRNA (Ebert et al., 1995). Influence of HIF-1α on activity of glycolytic enzymes was confirmed in the following cell lines: HepG2, HeLa, and L cells (mouse fibroblast). It was found that the action of HIF-1α is mediated by two enzymes: phosphoglycerate kinase-1 and lactate dehydrogenase A; HIF-1α binds to genes encoding these glycolytic enzymes modulating their expression (Firth et al., 1994; Weidemann and Johnson, 2008). Further studies allowed for new glycolytic enzymes influenced by HIF-1α to be determined, namely, hexokinase 1, hexokinase 2 (Soni and Padwad, 2017), enolase 1, aldolase A, and phosphofructokinase L (Iyer et al., 1998).

The effect of HIF-1α on GLUT-4 is similar to that on GLUT-1 the glucose uptake is increased. Knockdown of HIF-1α causes severe reduction in insulin-stimulated glucose uptake in cultured skeletal muscle cells due to impaired mobilization of GLUT4 to the plasma membrane (Sakagami et al., 2014).

The substrate for TAC is acetyl-CoA, which is produced from the end product of glycolysis, pyruvate. This process is called pyruvate decarboxylation and is mediated by the pyruvate dehydrogenase (PDH) complex, whose first component enzyme is PDH E1α. Pyruvate dehydrogenase kinase 1 can suppress PDH E1α activity through its phosphorylation and in this manner inhibit pyruvate decarboxylation (Nagao et al., 2019). Kim et al. found that HIF-1 suppressed TAC, activating the gene encoding pyruvate dehydrogenase kinase 1 (Kim et al., 2006; Semenza, 2007).

Surprisingly, HIF-1α is stabilized not only in a hypoxemic environment but also in normoxemia by interleukin-1 (IL-1) and insulin (Stiehl et al., 2002; Sakagami et al., 2014). Expression of HIF-1α protein in cultured skeletal muscle cells, even under normoxemia, was increased by stimulation with insulin for half an hour and remained elevated for at least the next 2 h (Sakagami et al., 2014). In some cell lines, hypoxia activates the phosphatidylinositol 3-kinase (PI3K)/Akt pathway (Chen et al., 2001), which also involved insulin signaling. The PI3K/Akt signaling pathway in HepG2 cells seems to be essential in HIF-1α response to hypoxia, insulin, and IL-1 due to its role in HIF-1α accumulation and stabilization (Stiehl et al., 2002).

Results from the Sato et al. (2014) research on mouse insulinoma cells (MIN6) provides information that hypoxia is responsible for transition of glucose metabolism from an oxidative to a glycolytic pathway, which consequently leads to decreased production of ATP in those cells. Investigating insulin secretion by MIN6 cells, insulin level in these cells was similar under either normoxic or hypoxic conditions. However, high glucose stimulation of MIN6 cells caused threefold higher insulin production under normoxia than under hypoxia. Furthermore, insulin secretion of MIN6 cells slightly decreased in normoxia compared to hypoxia in response to low glucose stimulation. The same study provides the data suggesting that hypoxia can lead to the downregulation of selected genes, which play important roles in β-cell function: *Foxa2, Mafa, Ins1, Neurod1, Pdx1, Wfs1, Slc2a2, Kcnj11*, and *Ndufa5* in both mouse islets and MIN6 cells; however, majority of the hypoxia-induced gene downregulations in cells were not related to HIF-1α suppression, suggesting a HIF-1α-autonomous mechanism (Sato et al., 2014).

Some studies showed that cells cultured in high glucose concentration medium present with decreased levels of HIF-1α. This led to a consensus that hyperglycemia was responsible for decreased HIF-1α protein levels (Xiao et al., 2013; Cerychova and Pavlinkova, 2018). Investigation of the effect of certain glucose concentrations on HIF-1α expression in human dermal fibroblasts (HDF) at normoxia and hypoxia showed that HIF-1α expression depends on glucose concentration only in hypoxia. At normoxia, no HIF-1α protein could be detected by Western blot analysis of HDF cell extracts and exposure to high glucose concentrations had no influence on HIF-1α expression. In the cells under hypoxic conditions, expression was decreasing gradually with the growing glucose concentrations of 5.5, 11, 25, and 30 mmol/l. Thus, the process of hypoxia-regulated stabilization of HIF-1α interferes with exposure of HDFs to high glucose concentrations (Catrina et al., 2004).

Although many studies support the thesis that hypoxia accompanied by HIF-1α overexpression is harmful to metabolism, there are also reports that suggest the beneficial influence of HIF-1α stabilization on glucose and lipid metabolism (Mackenzie et al., 2012; Jun et al., 2013; Mesarwi et al., 2015; Thomas et al., 2017). The study of Görgens et al. (2017) on human skeletal muscle cells reports that hypoxia in combination with muscle activity improved glucose metabolism and insulin activity via the HIF-1α and its influence on RAB20 and TXNIP transcription. Rab20 is a member of the Rab family of proteins, regulating intracellular trafficking and vesicle formation (Hackenbeck et al., 2011). Deletion of RAB20 impairs insulin-stimulated glucose uptake by blocking the translocation of GLUT4 to the cell surface. TXNIP encodes a thioredoxin-binding protein that is a member of the alpha arrestin protein family, which, among other functions, also regulates cellular metabolism (Shalev, 2014). TXNIP has been found to enhance insulin secretion and glucagon-like peptide 1 (GLP-1) signaling via regulation of a microRNA (Alhawiti et al., 2017; Thielen and Shalev, 2018). Under conditions applied in the study of Görgens et al. (2017) simulating physical exertion and hypoxia, RAB20 upregulation and TXNIP downregulation mediated by HIF-1α were detected in the investigated tissues, which may explain the beneficial influence in this case. These results suggest that HIF-1α stabilization in the combined setting of muscle contraction and hypoxia can counteract the development of IR (Görgens et al., 2017).

## Impact of HIF-1α on IR and T2DM in Animal Models

Glucagon-like peptide 1 is a hormone belonging to the incretin group, which enhances glucose-stimulated insulin secretion in β-cells (Carlessi et al., 2017) and suppresses glucagon secretion (Seino et al., 2010). Dipeptidyl peptidase-4 (DPP-4) is a multi-purpose protein, and one of its functions is the degradation of GLP-1, which leads to a decrease in endogenous GLP-1 levels (Deacon, 2019). Levels of active GLP-1 in T2DM are decreased (Vilsbøll et al., 2001; Holst et al., 2011). It was shown on a mice model that obesity reduces the level of active GLP-1 in peripheral circulation with increased level of DPP4, which leads to impaired glucose tolerance. Hepatocyte-specific HIF-1α knockout in mice blocked these changes induced by obesity (Lee et al., 2019).

Similar findings were disclosed in adipose tissue. To assess the influence of HIF-1α on the progression of obesity-induced diabetes in adipocytes, HIF-1α mRNA expression and GLP-1 levels were measured in epididymal adipose tissues of mice with and without HIF-1α knockout. The findings suggested that the knockout of HIF-1α in adipocytes increases glucose tolerance by enhancing insulin secretion through the increased GLP-1 levels (Kihira et al., 2014). The other known action of GLP-1 is induction of the expansion of β-cell mass responsible for insulin secretion, which results in the augmentation of glucose-stimulated insulin secretion (MacDonald et al., 2002). It was reported that deletion of HIF-1α in adipose tissue also ameliorates IR, which implies that HIF-1α could provide a novel potential therapeutic target for T2DM (Jiang et al., 2011).

The hypoxia and HIF-1α stabilization are also involved in the promotion of tissue inflammation, which further contributes to IR and T2DM development. With the onset of obesity, the adipose tissue becomes hypoxic (Ota et al., 2019). Various mechanisms thereof were suggested. The oxygen demand is increased due to uncoupled respiration in adipocytes (Lee et al., 2014). The capillary density is decreased and the perfusion of adipose tissue is reduced in obese patients, which makes oxygen delivery difficult and leads to hypoxia (Pasarica et al., 2009; Fujisaka et al., 2013). Furthermore, in obesity the oxygen diffusion in adipose tissue is less effective due to increased diameter of the cell (Lee et al., 2014). In response to tissue hypoxia, HIF-1α stabilization occurs. HIF-1α and NF-κB are involved in enhancing the inflammatory pathways in adipocytes, which leads to IR in the adipose tissue and other metabolic disturbances (Ota et al., 2019). Hypoxia-induced adipose tissue inflammation is characterized by the infiltration of classically activated macrophages M1. Macrophage phenotype is affected by HIF-1α-dependent and HIF-1α-independent pathways (Fujisaka et al., 2013). Interestingly, in the study performed on mice exposed to high-fat diet and the chronic intermittent hypoxia during sleep, it was reported that resveratrol administration may be beneficial in normalizing inflammatory process mediated by HIF-1α, leading to restoration of insulin responsiveness (Carreras et al., 2015).

Sacramento et al. (2016) exposed rats during sleep to hypoxic cycles for 28 or 35 days while the control group slept in normoxic conditions. After exposure to hypoxia, IR and fasting insulinemia increased along with chronic intermittent hypoxia duration, being significantly higher after exposure of 35 days. Additionally, chronic intermittent hypoxia decreased phosphorylation and expression of insulin receptor in adipose tissue and skeletal muscles, but not in the liver. Conversely, expression of GLUT-2 in the liver of animals exposed to chronic intermittent hypoxia was increased. Thirty-five days of chronic intermittent hypoxia also caused changes in the HIF-1α levels. HIF-1α upregulation was found in the liver cells, while it was downregulated in skeletal muscles (Sacramento et al., 2016). In another study, mice with partial deletion of HIF-1α were exposed to hypoxia for 8 h daily for the period of 2 weeks. Regardless of the partial HIF-1α deletion, the IR was increased in mice exposed to hypoxia in majority of tissues, suggesting the limited role of HIF-1α in hypoxia-induced IR (Thomas et al., 2017). Unexpectedly, in the same study, hypoxia induced improvement of glucose tolerance. This might be caused by muscle-specific stimulation of the AMPK-AS160/TBC1D1 signaling, which plays an important role in the regulation of glucose uptake (Thomas et al., 2017).

Sodium-glucose cotransporter 2 (SGLT2) inhibitors are a new group of medications used for treating T2DM; their action is based on reduction of glucose reabsorption, targeting the proximal tubules of nephrons. Bessho et al. (2019) treated male diabetic mice with SGLT2 inhibitor for 8–16 weeks. The results showed reduced cortical tubular HIF-1α expression followed by decreased tubular injury in mice. This implies SGLT2 inhibitors’ effect on diabetic mice (Bessho et al., 2019).

## HIF-1α in OSA Patients; IR, T2DM, and Its Complications

### HIF-1α in OSA Patients

To date, only few studies evaluated HIF-1α expression in OSA patients. Increased level of HIF-1α level in serum was observed in OSA patients compared to controls, regardless of measurement method (ELISA/western blot) (Lu et al., 2016; Gabryelska et al., 2019). Lack of difference between evening and morning concentrations suggests its chronic increasement caused by intermittent nocturnal hypoxia among OSA patients (Gabryelska et al., 2020c). Furthermore, one-night of CPAP therapy seems to be not sufficient to affect the increased level of the protein (Gabryelska et al., 2020b). On the other hand, Lu et al. (2016) observed decreased level of HIF-1α following 2 months of CPAP treatment compared to baseline results. In another study, Kaczmarek et al. (2013) examined skin biopsies from OSA patient (AHI ≥ 10) and found significant differences in *HIF-1*α mRNA expression level between groups with minimal oxygen saturation during PSG above or below 75%. Possibly showing that oxygen blood saturation might be the curtail factor for *HIF-1*α mRNA expression (aside from AHI as groups were matched regarding this variable), especially in tissues such as skin, which is more prone to hypoxia (Kaczmarek et al., 2013).

**FIGURE 1:**
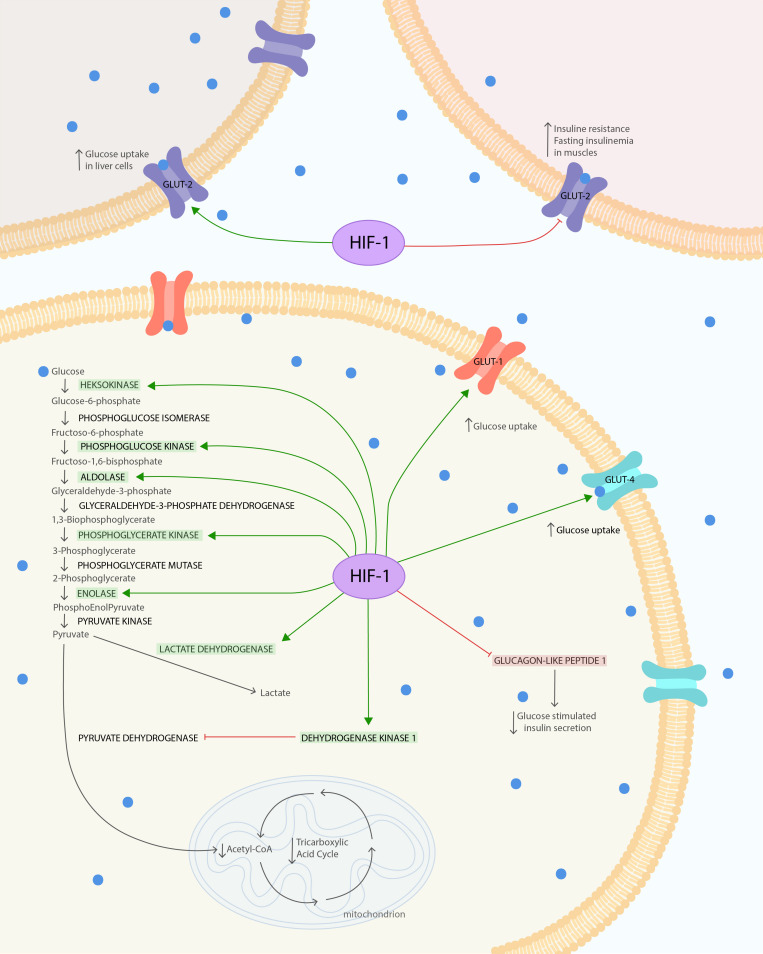
HIF-1α influence on glucose metabolism and insulin resistance. The HIF is composed of both oxygen-regulated a-subunit and constitutively expressed b-subunit. HIF-1α protein is highly unstable under normoxia condition. Hypoxia leads to stabilization of HIF-1α. HIF-1α under hypoxic conditions causes several changes in glucose metabolism. It increases glucose uptake via glucose transporters GLUT-1 and GLUT-4 to cells. In the cell, glucose is used in glycolysis, which is also enhanced due to the modulated expression of glycolytic enzymes: phosphoglycerate kinase-1, hexokinase 1, hexokinase 2, aldolase A, enolase 1, and phosphofructokinase L. The final product of glycolysis–pyruvate–is mostly converted to lactate instead of acetyl-CoA, due to the increased lactate dehydrogenase A activity and pyruvate decarboxylation inhibition. Pyruvate dehydrogenase kinase 1 (PDK1) action leads to suppression of dehydrogenase complex (PDH) through its phosphorylation and thereby inhibits pyruvate decarboxylation. Secondary to the decreased levels of acetyl-CoA and the action of PDK1, the TAC is downregulated. At the same time, the increased expression of HIF-1α reduces GLUT-2 phosphorylation and its expression in skeletal muscles. This leads to increased IR and fasting insulinemia after exposure to chronic hypoxia. To compensate for this metabolic imbalance, the expression of GLUT-2 in liver is increased. The glucose tolerance can also be impaired by upregulation of HIF-1α, leading to GLP-1 downregulation, which causes reduction in glucose-stimulated insulin secretion via pancreatic b-cells. The Figure 1 was prepared in Adobe Illustrator (Adobe Inc., San Jose, CA, United States).

### Effects of HIF-1α on IR, TD2M, and Its Complications in Humans

The level of serum HIF-1α was found to be significantly increased in patients suffering from T2DM compared to the control group (Shao et al., 2016). Furthermore, the presence of the non-synonymous single-nucleotide polymorphism (rs11549465) in HIF-1α gene in the Japanese and Hungarian populations reduced the risk of developing diabetes (Geza et al., 2009). Additionally, some reports suggested that HIF-1α is involved in development of T2DM complications. Diabetic foot ulcers (DFU) are among very frequent complications of diabetes mellitus, especially when disease is not well controlled (Yazdanpanah et al., 2015). DFU develops as a consequence of a combination of factors: peripheral neuropathy, peripheral vascular disease, and trauma (Boulton and Whitehouse, 2000). It has been shown that biopsy samples obtained from the margin of chronic DFU express decreased HIF-1α levels compared to samples from the margin of chronic venous ulcers (Catrina et al., 2004; Catrina and Zheng, 2016). Faint HIF-1α staining in DFU, similar to the staining pattern characteristic of exposure to the normoxic conditions, was found. In contrast, positive HIF-1α staining, like in cells under hypoxic conditions, was identified in both the nuclei and cytoplasm in majority of fibroblasts and a few endothelial cells in venous ulcers. It may suggest an important involvement of hyperglycemia in control of HIF1-α protein levels in tissues under hypoxia (Catrina et al., 2004). Unfortunately, wounds in DFU heal poorly. The reason for that phenomenon can be compromised blood vessel formation in response to ischemia and hyperglycemia (Thangarajah et al., 2010). This impairment in vascularization can result from hyperglycemia-induced inhibition of HIF-1α, which is transcription factor regulating the expression of vascular endothelial growth factor (VEGF). Deferoxamine (DFO) is a drug that may reverse the effect of HIF1-α inhibition (Thangarajah et al., 2010). DFO is an iron ion chelator-antioxidant. The main indication for DFO treatment is diseases with iron overload such as hemosiderosis (Di Nicola et al., 2015). However, DFO can also upregulate HIF-1α via triggering the ERK signaling pathway (Guo et al., 2016). HIF-1α upregulation accelerates the recovery process of humanized diabetic wounds in animal models. It suggests that HIF1-α can be the target of therapy in this very common T2DM complication (Thangarajah et al., 2010). There is a clinical trial pending, which investigates the effect of local DFO (0.66 mg/ml) treatment on the wound healing process in patients with DFU. The main endpoint of this trial will be to reduce more than 50% the wound area after 12 weeks of DFO treatment (ClinicalTrials.gov: NCT03137966). The other drug increasing HIF1-α expression in DFU being tested in clinical trials is pirfenidone (PFD) applied with modified diallyl disulfide oxide (M-DDO). PFD indication is treatment of idiopathic pulmonary fibrosis due to its antifibrogenic action. M-DDO is an antimicrobial and antiseptic agent. However, their combined administration can influence the gene expression and increase HIF1-α action (ClinicalTrials.gov: NCT02632877) (Gasca-Lozano et al., 2017).

Another common complication of T2DM is diabetic retinopathy. Abnormal retinal blood vessel growth and diabetic macular edema are two crucial problems causing vision loss in diabetic patients (Crawford et al., 2009). Increased levels of HIF-1α in hypoxia are significantly related to retinal angiogenesis responsible for abnormal retinal blood vessels growth. Suppression of HIF-1α reduced VEGF expression and can prevent unwanted angiogenesis. This phenomenon suggests that HIF-1α may be a target in pharmacological treatment for diabetic retinopathy (Cheng et al., 2017; Zhang et al., 2018).

## Conclusion

Available literature shows that HIF-1α is involved in regulation of metabolic processes and mediates development of IR and diabetes mellites. However, vast majority of the studies are based on cellular and animal models of hypoxia. As few available studies show that HIF-1α in OSA patients is upregulated, it is probable that HIF-1α might be involved in development of metabolic comorbidities in this group. Nevertheless, further studies are needed to support this plausible pathomechanism. Taking under consideration the fact that animal studies suggest HIF-1α as a possible therapeutic target in impaired glucose metabolism, this might be a promising research direction in OSA patients.

## Author Contributions

AG created the concept of the manuscript. AG, FK, and BS conducted the literature research and wrote the manuscript. PB revised the manuscript.

## Conflict of Interest

The authors declare that the research was conducted in the absence of any commercial or financial relationships that could be construed as a potential conflict of interest.

## Abbreviations

AHI: apnea–hypopnea index
CPAP: continuous positive air pressure
DFO: deferoxamine
DFU: diabetic foot ulcers
DPP-4: dipeptidyl peptidase-4
GLP-1: glucagon-like peptide-1
GLUT-1: glucose transporter type 1
GLUT-2: glucose transporter type 2
GLUT-4: glucose transporter type 4
HDF: human dermal fibroblasts
HIF: hypoxia-inducible factors
HIF-1 α: α-subunit of hypoxia-inducible factor 1
HIF-2 α: α-subunit of hypoxia-inducible factor 2
IL-1: interleukin-1
IR: insulin resistance
M-DDO: modified diallyl disulfide oxide
OSA: obstructive sleep apnea syndrome
PDH: pyruvate dehydrogenase complex
PFD: pirfenidone
PI3K: phosphatidylinositol 3-kinase
PSG: polysomnography
SGLT2: sodium-glucose cotransporter 2
T2DM: type 2 diabetes
TAC: tricarboxylic acid cycle
VEGF: vascular endothelial growth factor.
